# Longitudinal analyses of serum neurofilament light and associations with obesity indices and bioelectrical impedance parameters

**DOI:** 10.1038/s41598-022-20398-y

**Published:** 2022-09-23

**Authors:** Marco Hermesdorf, David Leppert, Aleksandra Maceski, Pascal Benkert, Jürgen Wellmann, Heinz Wiendl, Jens Kuhle, Klaus Berger

**Affiliations:** 1 Institute of Epidemiology and Social Medicine, University of Münster, Albert-Schweitzer-Campus 1, 48149 Münster, Germany; 2 Multiple Sclerosis Centre, Neurology, Departments of Head, Spine and Neuromedicine, Biomedicine and Clinical Research, University Hospital Basel and University of Basel, Basel, Switzerland; 3 Research Center for Clinical Neuroimmunology and Neuroscience (RC2NB), University Hospital and University of Basel, Basel, Switzerland; 4 Department of Clinical Research, University Hospital Basel, University of Basel, Basel, Switzerland; 5 Department of Neurology with Institute of Translational Neurology, University Hospital Münster, Münster, Germany

**Keywords:** Biomarkers, Neuroscience, Neurology

## Abstract

Neurofilament light is a constituent of the neuronal cytoskeleton and released into the blood following neuro-axonal damage. It has previously been reported that NfL measured in blood serum is inversely related to body mass index. However, no reports exist with regard to body composition assessed using bioelectrical impedance analysis or other indicators of obesity beyond BMI. We analyzed the relationship between sNfL and body composition according to the three compartment model. Additionally, associations between sNfL, body shape index, waist-to-height ratio, and BMI were examined. The sample consisted of 769 participants assessed during the baseline examination and 693 participants examined in the course of the follow-up of the BiDirect Study. Associations between sNfL, BMI, BSI, and WtHR were separately analyzed using linear mixed models. Body compartments operationalized as fat mass, extracellular cell mass, and body cell mass were derived using BIA and the relationship with sNfL was analyzed with a linear mixed model. Lastly, we also analyzed the association between total body water and sNfL. We found significant inverse associations of sNfL with BMI and WtHR. The analysis of the three compartment model yielded significant inverse associations between sNfL, body cell mass and body fat mass, but not extracellular mass. Furthermore, total body water was also inversely related to sNfL. A potential mechanism could involve body cell mass and body fat mass as highly adaptive body constituents that either directly absorb sNfL, or promote the formation of new vasculature and thereby increase blood volume.

## Introduction

Neurofilament light polypeptide (NfL) is a protein found in large-caliber axons and a constituent of the neuronal cytoskeleton^1^. When neuroaxonal damage occurs and white matter disintegrates, NfL is released into the extracellular space from where it reaches the cerebrospinal fluid (CSF) and the blood stream^2^. Previous studies have shown that NfL levels are increased in several neurological disorders, e.g. Alzheimer’s disease^3^, traumatic brain injury^4^, or multiple sclerosis^5^. Therefore, NfL is considered a general biomarker for neuroaxonal damage and disease activity^6^, particularly since NfL levels can be measured in the blood serum without exposing an individual to the risks of a lumbar puncture. The minimally invasive assessment of serum NfL (sNfL) makes it feasible for the application in large population-based studies.

Previous studies analyzed associations between sNfL and potential confounders in order to improve the interpretability of sNfL levels for the application in clinical settings. These studies revealed that sNfL increases with age in the general population^5,7^ and that sNfL is inversely related to body mass index (BMI) and body blood volume^8^. The inverse association between sNfL, BMI, and blood volume is particularly interesting because it is only observed if NfL is measured in the blood but not in the CSF. BMI as a surrogate marker of over- and underweight is a well-established risk factor^9^ for several adverse disease outcomes and, thus, might be considered a confounder. The etiology of this association is not fully understood.

The aims of this study were to analyze the associations between sNfL and several anthropometric indices operationalized as BMI, body shape index (BSI), and waist-to-height ratio (WHtR). BMI does not discriminate^10^ between body fat and fat free mass (e.g. skeletal muscle) whereas BSI and WtHR are better indicators of unhealthy body fat distribution^11^. It was hypothesized that BSI and WHtR exhibit an inverse association with sNfL comparable to the already known relationship with BMI. Additionally, we explored associations between sNfL and body composition parameters estimated by bioelectrical impedance analysis (BIA), particularly body fat mass (BFM), body cell mass (BCM), extracellular mass (ECM), and total body water (TBW).

## Results

**Figure 1:**
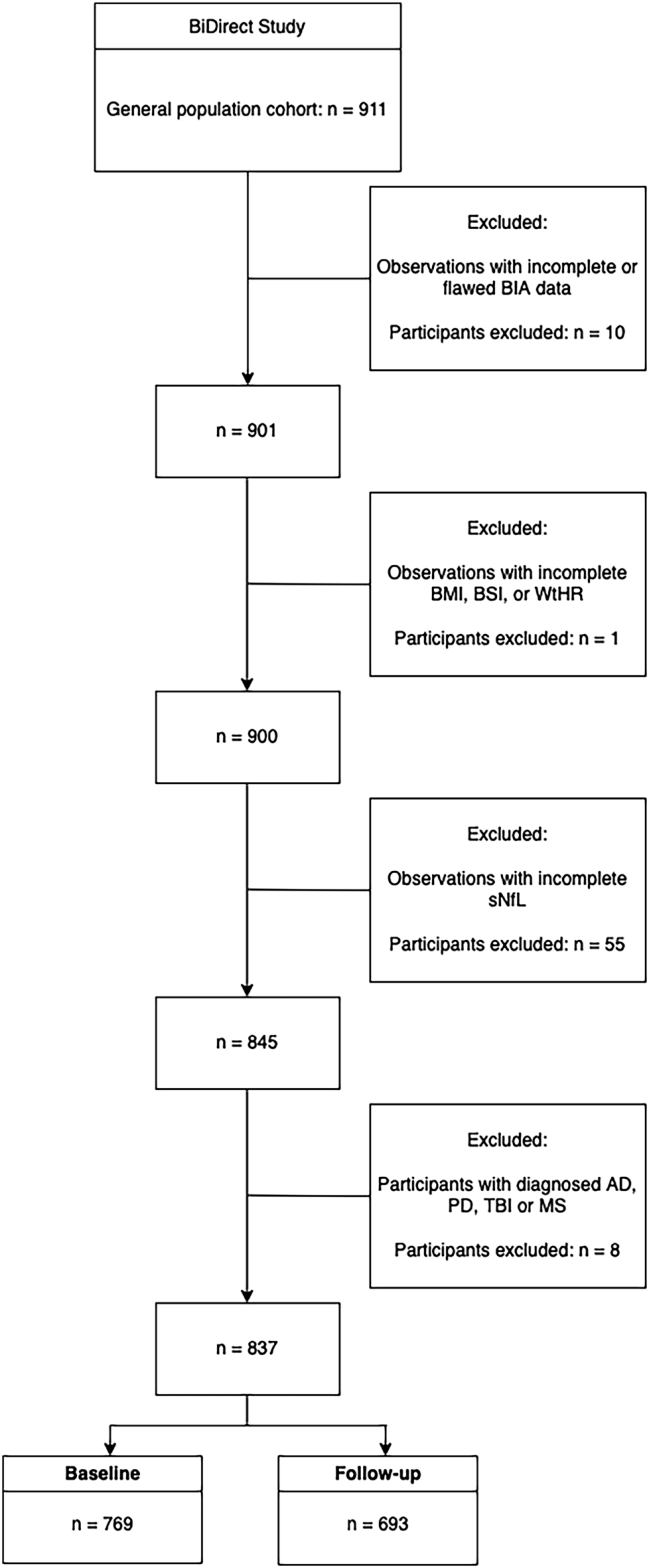
Flowchart illustrating the exclusion criteria and the number of participants considered for the analyses of both time points. *Abbreviations*: BIA, bioelectrical impedance analysis; BMI, body mass index; BSI, body shape index; WtHR, waist-to-height ratio; sNfL, serum neurofilament light; AD, Alzheimer’s disease; PD, Parkinson’s disease, TBI, traumatic brain injury; MS, multiple sclerosis.

**Figure 2:**
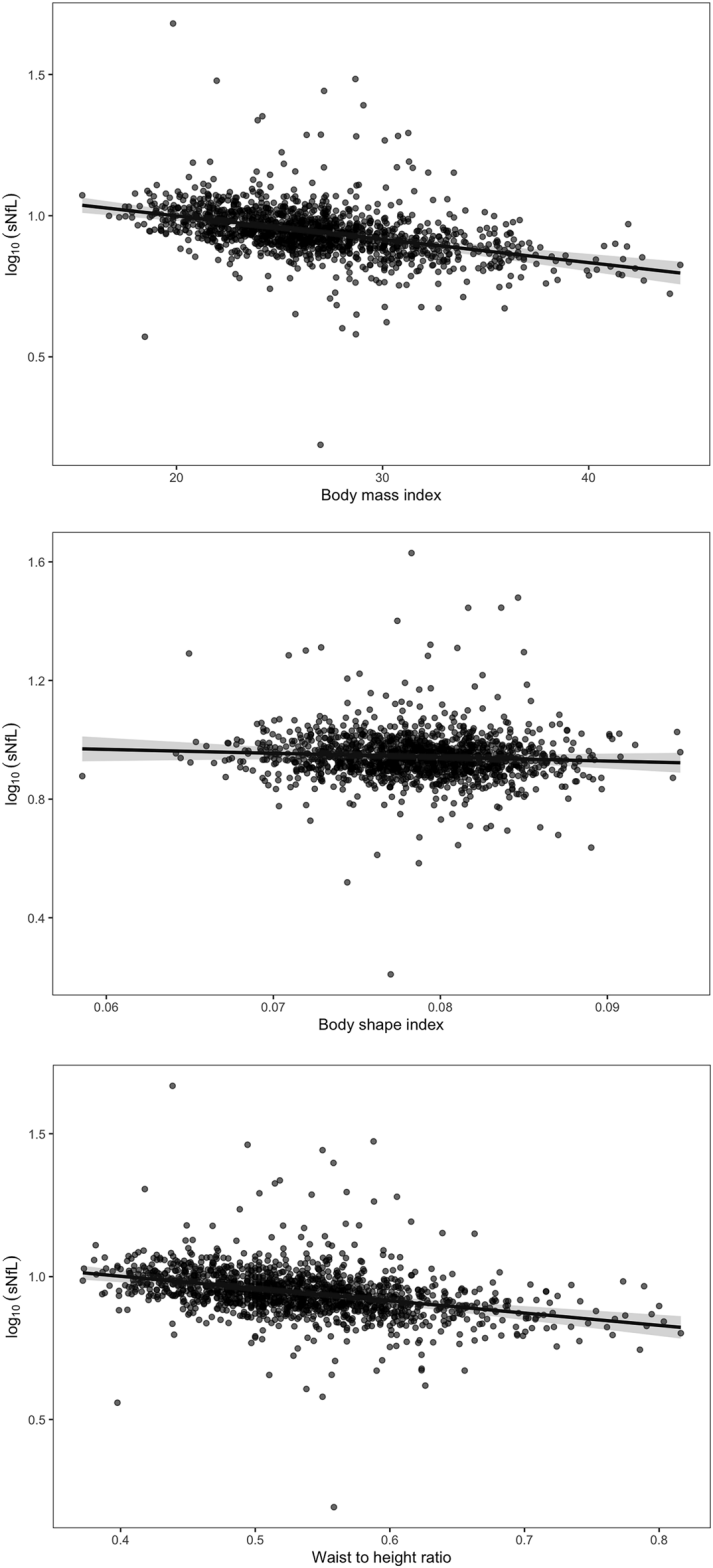
Partial residual plots showing the sex- and age-adjusted, separate associations of sNfL with body mass index, body shape index, and waist-to-height ratio. The grayish shaded areas indicate the 95% confidence intervals.

**Figure 3:**
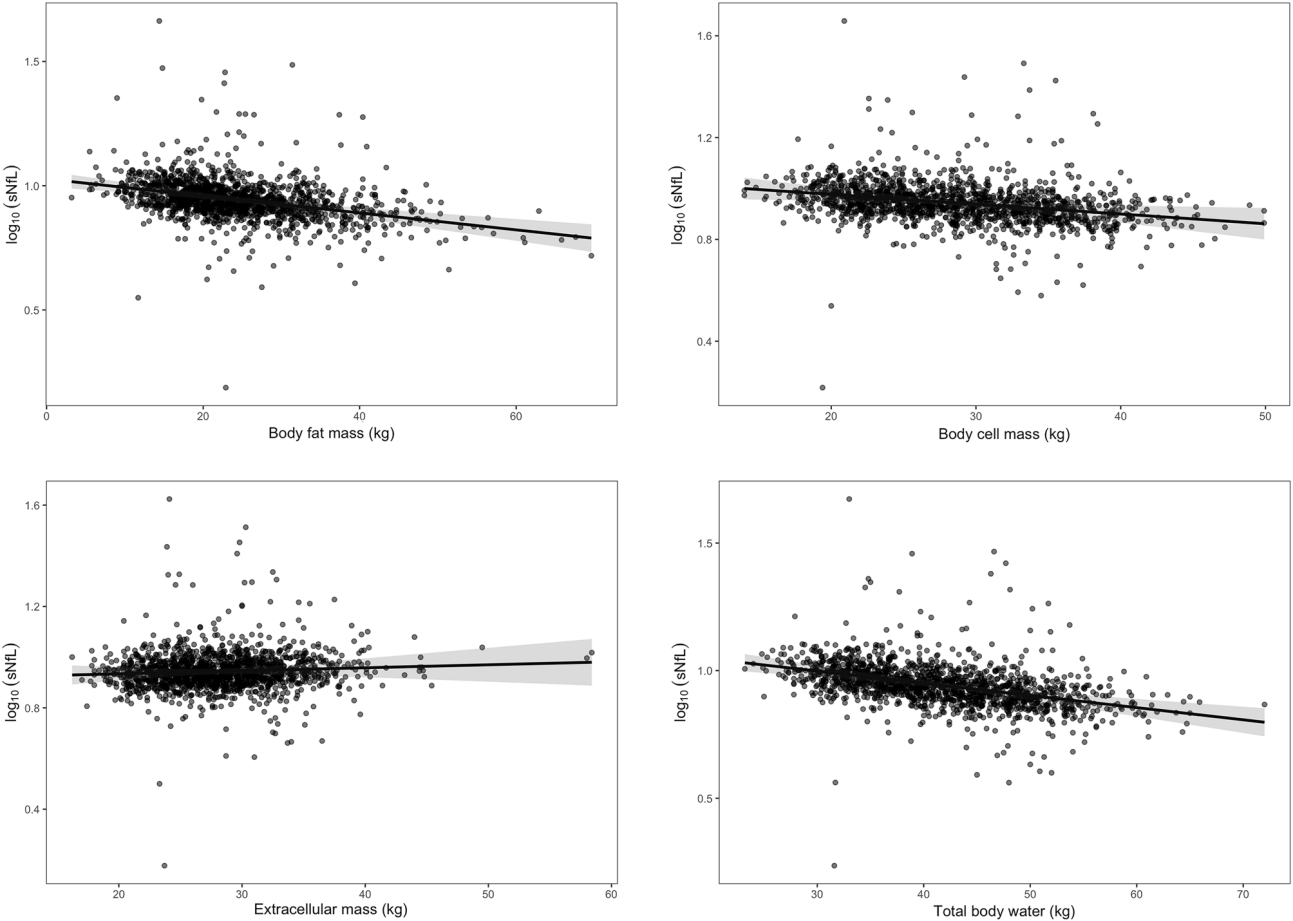
Partial residual plots showing the sex- and age-adjusted inverse associations between sNfL and body fat mass, and extracellular mass, and body cell mass according to the three-compartment model. The bottom right panel shows the sex- and age-adjusted inverse association between sNfL and total body water. The 95% confidence intervals are shown as grayish shaded areas.

The exclusion criteria and sample characteristics for both time points are summarized in Fig. 1 and Table 1, respectively. In participants (n = 625) who provided data to both time points, sNfL increased by a mean of 0.95 pg/ml over an average of 2.7 years. The initial linear model for the cross-sectional baseline data showed a significant inverse association between sNfL and BMI (*β* = − 0.009, 95% confidence interval = − 0.012 to − 0.007, *p* < 0.001). The separate longitudinal analyses of associations between sNfL and the three anthropometric indices of obesity revealed that BMI (*β* = − 0.008, 95% confidence interval = − 0.01 to − 0.006, *p* < 0.001, semi-partial *R*^2^ = 0.034) and WtHR (*β* = − 0.43, 95% confidence interval = − 0.56 to − 0.30, *p* < 0.001, semi-partial *R*^2^ = 0.024) were inversely related to sNfL, whereas the association with BSI was not statistically significant (*β* = − 1.31, 95% confidence interval = − 3.33 to 0.72, *p* = 0.205, semi-partial R^2^ = 0.001). Partial residual plots showing the adjusted associations with the anthropometric indices are shown in Fig. 2. The analysis of the three-compartment model yielded significant associations with BCM and BFM, as shown in Table 2 and Fig. 3, but not with ECM. Furthermore, TBW (*β* = − 0.005, 95% confidence interval = − 0.007 to − 0.003, *p* < 0.001, semi-partial *R*^2^ = 0.015) was significantly and inversely related to sNfL.

**Table 1:** Demographic characteristics of the sample.

	Time point 1 (n = 769)	Time point 2 (n = 693)
Age (years): mean (SD)	52.78 (8.11)	55.86 (8.01)
Women: n (%)	392 (51)	350 (50.1)
sNfL (pg/ml): median (IQR)	8.2 (4.3)	9.1 (4.8)
Log-10 sNfL: mean (SD)	0.92 (0.19)	0.97 (0.19)
Weight (kg): mean (SD)	80.01 (16.48)	79.25 (15.53)
Height (m): mean (SD)	1.73 (0.09)	1.72 (0.09)
Waist circumference (m): mean (SD)	0.92 (0.13)	0.92 (0.13)
BMI: mean (SD)	26.71 (4.5)	26.63 (4.37)
BSI: mean (SD)	0.08 (0.005)	0.08 (0.004)
WtHR: mean (SD)	0.53 (0.07)	0.54 (0.07)
BFM (kg): mean (SD)	24.18 (9.20)	24.62 (8.83)
ECM (kg): mean (SD)	28.18 (5.03)	28.05 (4.97)
BCM (kg): mean (SD)	29.02 (6.56)	27.96 (6.38)
TBW (kg): mean (SD)	41.87 (7.97)	41.0 (7.76)

*SD* standard deviation, *sNfL* serum neurofilament light, *BMI* body mass index, *BSI* body shape index, *WtHR* waist-to-height ratio, *BFM* body fat mass, *ECM* extracellular mass, *BCM* body cell mass, *TBW* total body water.

**Table 2:** Serum neurofilament light and body composition.

Model 1	*β* (95% CI)	*p*	Semi-partial *R*2
Age	0.012 (0.011 to 0.013)	< 0.001	0.2307
Sex	0.017 (− 0.016 to 0.049)	0.313	0.0002
BFM	− 0.003 (− 0.005 to − 0.002)	< 0.001	0.0234
ECM	0.001 (− 0.002 to 0.004)	0.44	0.0000
BCM	− 0.004 (− 0.007 to − 0.001)	0.008	0.0029

Nakagawa's conditional R2 = 0.72; Nakagawas marginal R2 = 0.31.

*CI* confidence interval, *BFM* body fat mass, *ECM* extracellular mass, *BCM* body cell mass.

## Discussion

The present study analyzed associations between sNfL, anthropometric indices, bioelectrical impedance parameters according to the three-compartment model, and TBW. The results showed significant inverse associations between sNfL levels and BMI, WtHR, BCM, BFM, and TBW. However, no significant estimates were obtained for BSI and ECM.

Our results replicated earlier findings regarding the inverse relationship between sNfL and BMI^5,8^, and extended these findings to anthropometric indices such as the WtHR. The latter is a better indicator of unhealthy body fat distribution^11^. However, the BSI is also an indicator of unhealthy body fat distribution but was not significantly associated with sNfL. These findings were complemented by the BIA, showing an inverse association between sNfL, BFM, and BCM. This relationship could be explained by the role of adipose tissue and BCM in angiogenesis and blood volume. BCM, which includes highly adaptive and metabolically active tissue^12^ (e.g. skeletal muscle), is related to the formation of new blood vessels depending on the level of exercise and can increase the circulating blood volume^13,14^. This contrasts with ECM, which is metabolically inactive and less adaptive compared to BCM^15^. It has also been observed that expanding adipose tissue is paralleled by increased blood vessel formation^16^, which consequently manifests in larger blood volume^17^. Thus, it is suggested that BFM and metabolically active BCM^12^ may be related to increased blood volume thereby decreasing the concentration of proteins of neuronal origin, or a direct distribution into tissue. This perspective is also supported by the inverse relationship between sNfL and TBW, and the previously observed inverse association between sNfL and blood volume^8^, calculated based on weight and height^18^. The current results from the BIA support and extend these previous observations. Apart from sNfL, previous studies that employed blood-based analyses using ultrasensitive assays have observed similar inverse associations between BMI and proteins originating from the brain. In particular, an inverse association between standardized BMI and glial fibrillary acidic protein (GFAP) levels has been reported recently^19^. An inverse relationship between BMI and phosphorylated tau protein^20^ has also been observed, although there is conflicting evidence^21^.

Together with previous findings, the present results suggest attenuated sNfL concentrations related to body composition, captured by ultrasensitive assays measuring proteins of neuronal origin in the blood. Future studies analyzing sNfL should therefore consider to include BMI (or other indicators of body composition) in their statistical models as an easy to measure confounder, e.g. in the field of dementia where it has been observed that BMI declines over time prior to the clinical onset of Alzheimer’s disease^22^, or apply age and BMI adjusted percentile scores^5^ for individual application. Further studies analyzing the relationship between body composition and attenuated concentrations of proteins of neuronal origin measured in the blood are required.

The BMI does not allow a distinction between body fat and other body constituents^10^ and does not capture sarcopenia^23^, a type of muscle loss associated with aging. However, a fine-grained assessment of body composition using BIA can overcome these limitations by distinguishing between BCM, BFC, and ECM. Although body composition can be even more precisely assessed using other methods, body composition estimates derived with BIA are in good agreement with those derived by computer tomography or hydrostatic weighting^23,24^, which are unfeasible to conduct in larger samples. A major strength of our study is the large, population-based sample with repeated assessments of BIA parameters and anthropometric indicators of obesity over the course of 2.7 years. However, the age range of the current sample was limited between 35 and 65 years during recruitment and a steeper association between sNfL and age has been observed in older samples^5,7^. We were not able to provide results for other body indices such as the waist-to-hip ratio because hip circumference was not assessed in the course of the study.

In conclusion, we observed an inverse association between sNfL and body composition, particularly BCM, BFM, and TBW, suggesting a compartment-specific attenuation of neuronal biomarkers captured by ultrasensitive assays.

## Methods

### Participants

The participants were examined in the course of the longitudinal BiDirect Study^25^, which includes two patient cohorts and a cohort of population-based controls. The latter cohort was invited to participate after taking a random sample based on the local population register of the city of Münster (Germany), and selected for the present analyses. The population-based cohort included 911 participants aged between 35 and 65 during recruitment. We considered those subjects who participated in the baseline and/or first follow-up assessment where they underwent BIA. Participants who did not undergo BIA or with any missing values were excluded on the survey level. One observation with flawed BCM data (below zero) as well as implausible ECM (above 110 kg) was also excluded from the analyses. This resulted in 837 unique subjects, as shown in Fig. 1, where 769 of participated in the baseline and 693 in the follow-up examination. A total of 625 (75%) participants took part in both examinations. Written informed consent was obtained from all participants. The BiDirect Study was conducted in accordance with the declaration of Helsinki and approved by the ethics committee of the Medical Faculty of the University of Münster.

### Anthropometric indices and bioimpedance analysis

Participants’ weight (kg), height and waist circumference (m) were assessed in a standardized manner in the course of the comprehensive physical examination program of the BiDirect Study. BMI ($\frac{weight}{{height^{2} }}$), BSI ($$\frac{waist\,\, circumference}{{\begin{array}{*{20}c} {BMI^{\frac{2}{3}} height^{\frac{1}{2}} } \\ \\ \end{array} }}$$), and WHtR ($\frac{waist\,\,circumference}{{height}}$) were calculated based on the respective measurements. Body composition was evaluated using a BIA 2000-S device (Data Input GmbH, Pöcking, Germany) and categorized into BCM, BFM, and ECM according to the three-compartment model^26–28^. The three-compartment model has proved beneficial over other models of body composition like the two-compartment model, which separates the body into BFM and fat free mass but does not capture BCM^27,29^. Additionally considering BCM and ECM with the three-compartment model allows a reliable estimation of body composition using BIA^23^.

### Serum neurofilament light

Non-fasting blood samples were taken, centrifuged and stored at − 80 °C within two hours. Later, sNfL was quantified by blinded, board-certified technicians using a commercially available kit (NF-Light, Quanterix) and a single molecule array (SIMOA) HD-X analyzer (Quanterix, Lexington, MA, USA). The coefficients of variation were below 15% for all samples. Due to the right-skewed distribution of sNfL, the variable was log_10_-transformed prior to the analyses.

### Statistical analysis

In a first step, we aimed to replicate previously reported cross-sectional associations between sNfL as a dependent variable and BMI with a linear regression model for the baseline data, adjusted for age and sex. Next, longitudinal associations between sNfL as dependent variable and BMI, BSI and WtHR as respective independent variables were analyzed with three separate linear mixed models using the lmerTest package^30^ for R. Additional linear mixed models were run to investigate associations between sNfL and BCM, ECM and BFM according to the three-compartment model of body composition, and to examine associations between sNfL and TBW. All models were adjusted for age and sex. A random intercept was included in all longitudinal models to account for the correlated structure of repeated measurements over time. *P* values below 0.05 (two-tailed) were considered significant. All analyses were conducted with R version 4.1.2.

## Data Availability

The dataset used and analyzed during the current study is available from the corresponding author on reasonable request.

## References

[1] Lee MK, Cleveland DW (1996). Neuronal intermediate filaments. Annu. Rev. Neurosci..

[2] Petzold A (2005). Neurofilament phosphoforms: Surrogate markers for axonal injury, degeneration and loss. J. Neurol. Sci..

[3] Zetterberg H (2016). Association of cerebrospinal fluid neurofilament light concentration with Alzheimer disease progression. JAMA Neurol..

[4] Zetterberg H, Smith DH, Blennow K (2013). Biomarkers of mild traumatic brain injury in cerebrospinal fluid and blood. Nat. Rev. Neurol..

[5] Benkert P (2022). Serum neurofilament light chain for individual prognostication of disease activity in people with multiple sclerosis: A retrospective modelling and validation study. Lancet Neurol..

[6] Leppert D, Kuhle J (2019). Blood neurofilament light chain at the doorstep of clinical application. Neurol. Neuroimmunol. NeuroInflamm..

[7] Khalil M (2020). Serum neurofilament light levels in normal aging and their association with morphologic brain changes. Nat. Commun..

[8] Manouchehrinia A (2020). Confounding effect of blood volume and body mass index on blood neurofilament light chain levels. Ann. Clin. Transl. Neurol..

[9] Bhaskaran K, dos-Santos-Silva I, Leon DA, Douglas IJ, Smeeth L (2018). Association of BMI with overall and cause-specific mortality: A population-based cohort study of 3·6 million adults in the UK. Lancet Diabetes Endocrinol..

[10] Liu J, Tsilingiris D, Dalamaga M (2022). The non-linear relationship between muscle mass and BMI calls into question the use of BMI as a major criterion for eligibility for bariatric surgery. Metab. Open.

[11] Piché M-E, Poirier P, Lemieux I, Després J-P (2018). Overview of epidemiology and contribution of obesity and body fat distribution to cardiovascular disease: An update. Prog. Cardiovasc. Dis..

[12] Müller MJ (2013). From BMI to functional body composition. Eur. J. Clin. Nutr..

[13] Convertino VA (2007). Blood volume response to physical activity and inactivity. Am. J. Med. Sci..

[14] Olfert IM, Baum O, Hellsten Y, Egginton S (2016). Advances and challenges in skeletal muscle angiogenesis. Am. J. Physiol. Circ. Physiol..

[15] Weinreich T, Filz HP, Gresser U, Richartz BM (2017). Effectiveness of a four-week diet regimen, exercise and psychological intervention for weight loss. J. Clin. Diagn. Res..

[16] Christiaens V, Lijnen HR (2010). Angiogenesis and development of adipose tissue. Mol. Cell. Endocrinol..

[17] Gibson JG, Evans WA (1937). Clinical studies of the blood volume. Ii. the relation of plasma and total blood volume to venous pressure, blood velocity rate, physical measurements, age and sex in ninety normal humans. J. Clin. Invest..

[18] Nadler SB, Hidalgo JH, Bloch T (1962). Prediction of blood volume in normal human adults. Surgery.

[19] Hellerhoff I (2021). Differential longitudinal changes of neuronal and glial damage markers in anorexia nervosa after partial weight restoration. Transl. Psychiatry.

[20] Brickman AM (2021). Plasma p-tau181, p-tau217, and other blood-based Alzheimer’s disease biomarkers in a multi-ethnic, community study. Alzheimer’s Dement..

[21] Syrjanen JA (2021). Associations of amyloid and neurodegeneration plasma biomarkers with comorbidities. Alzheimer’s Dement..

[22] Gu Y (2014). Change in body mass index before and after Alzheimer’s disease onset. Curr. Alzheimer Res..

[23] Ceniccola GD (2019). Current technologies in body composition assessment: Advantages and disadvantages. Nutrition.

[24] Moon JR (2008). Percent body fat estimations in college men using field and laboratory methods: A three-compartment model approach. Dyn. Med..

[25] Teismann H (2014). Establishing the bidirectional relationship between depression and subclinical arteriosclerosis – rationale, design, and characteristics of the BiDirect Study. BMC Psychiatry.

[26] Talluri A, Maggia G (1995). Bioimpedance analysis (BIA) in hemodialysis: Technical aspects. Int. J. Artif. Organs.

[27] Siri WE (1956). Body composition from fluid spaces and density: Analysis of methods. Nutrition.

[28] Hamada Y (2015). Objective data assessment (ODA) methods as nutritional assessment tools. J. Med. Investig..

[29] Esco MR, Nickerson BS, Fedewa MV, Moon JR, Snarr RL (2018). A novel method of utilizing skinfolds and bioimpedance for determining body fat percentage via a field-based three-compartment model. Eur. J. Clin. Nutr..

[30] Kuznetsova A, Brockhoff PB, Christensen RHB (2017). lmerTest package: Tests in linear mixed effects models. J. Stat. Softw..

